# NDT of Residual Stress in Thick Aluminum Alloy Plates under Different Aging Conditions Using Multiple Techniques

**DOI:** 10.3390/ma15248732

**Published:** 2022-12-07

**Authors:** Zhengyi Li, Bing Xue, Yan Cui, Gang Zhou, Shaohua Zhang, Ning Lu, Lei Wen, Duzhou Zhang

**Affiliations:** 1 National Center for Materials Service Safety, University of Science and Technology Beijing, Beijing 100083, China; 2 Beijing Institute of Control Engineering, Beijing 100081, China; 3 The Second Gas Production Plant of Changqing Oilfield Company of CNPC, Yulin 719000, China; 4 China Academy of Space Technology, Beijing 100081, China

**Keywords:** synchrotron radiation diffraction, residual stress, cosα method, sin^2^ψ method, aluminum alloy

## Abstract

In this paper, a portable residual stress tester and synchrotron radiation diffraction technique were used to measure the residual stress distribution of thick 2A14 aluminum alloy plates under different aging conditions after solution treatment. The stress changes after solution and aging were analyzed using metallographic structure observation, electron backscattered diffraction (EBSD), X-ray diffraction (XRD), and other characterization methods. The results show that after solution treatment at 500 °C and aging at 170 °C, the second phase precipitates, lattice distortion is released, and the stress level gradually decreases with aging time. The residual stress of the plate comprises compressive stress; there is stress concentration in the central area. The stress distribution obtained by the two residual stress calculation methods, cosα and sin^2^ψ, under different process conditions was consistent.

## 1. Introduction

High-strength heat-resistant aluminum alloy is widely used in the aerospace field, and one form of it is 2A14 aluminum alloy. For greater toughness, 2A14 aluminum alloy must be solution-treated after rolling; this is because the rolling process induces great residual stress and affects the dimensional stability of the parts. In addition, residual stress and the eventual service environment will cause a series of adverse effects, such as deformation [1], fatigue damage [2], and stress corrosion [3,4]. Therefore, the residual stress and structure of aluminum alloy components during heat treatment were studied [5,6], the results of which have great significance for improving the machining accuracy and service life of materials.

Non-destructive testing (NDT) methods include the X-ray diffraction (XRD) method, synchrotron radiation method, neutron method, ultrasonic method, and magnetic strain method [7]. The XRD method is the most widely used NDT method [8], offering fast testing speed, but the disadvantage is that it demonstrates low brightness and poor penetration. In recent years, with the rapid development of synchrotron radiation technology, more and more attention has been paid to the measurement of residual stress [9].

Synchrotron radiation X-ray technology, which can quickly measure the internal residual stress [10,11,12,13,14], is particularly suitable for studying the residual stress in the near-surface area of the materials. Q. Wu [15] studied the cutting residual stress of 7075 aluminum alloy using XRD technology and the finite element simulation method and successfully simulated the stress distribution on the machined surface after three-dimensional milling. S. Ferreira-Barragáns [16] used the high-energy synchronous XRD method to test the residual stress of 2014 aluminum alloy, which was aged at 200 °C for different periods. The results showed that the stress gradually decreased with the increase in aging time. Tanner [17] studied the residual stress and mechanical properties of 2014 aluminum alloy at different cooling rates and found that the mechanical properties and residual stress of 2014 aluminum alloy were not greatly reduced when they were quenched at 60 °C. The homogeneity of the microstructure and mechanical properties of thick aluminum alloy plates is the key to the properties of aluminum alloy. At present, research on testing the residual stress of thick aluminum alloy plates via synchrotron radiation technology is sparse. The stress is not measured directly but is instead established using the calculation method; therefore, the accuracy of the stress measurement value depends on the stress measurement method. At present, there are two main methods that are used for residual stress determination by XRD: the sin^2^ψ method and the cosα method. The cosα method is not yet mature in terms of technology. For users examining complex stress situations in terms of parts and materials and high requirements of residual stress measurement, the sin^2^ψ method is more effective [18].

Therefore, the residual stress of 2A14 aluminum alloy was studied by XRD and synchrotron radiation diffraction, calculated using the cosα method and sin^2^ψ method, respectively. In this experiment, the residual stress distribution of aluminum alloy plate with different solutions and under various aging conditions was obtained, and the influence of different aging times on residual stress was analyzed.

## 2. Materials and Methods

### 2.1. Materials

The experimental material of 2A14 aluminum alloy plate was of four sizes: 100 mm × 100 mm × 50 mm, 100 mm × 100 mm × 30 mm, 100 mm × 100 mm × 20 mm, and 100 mm × 100 mm × 10 mm; the latter three sizes were only used for XRD analysis. It is a form of Al-Cu-Mg alloy, and its chemical composition, which was analyzed by XRF, is shown in Table 1.

**Figure 1:**
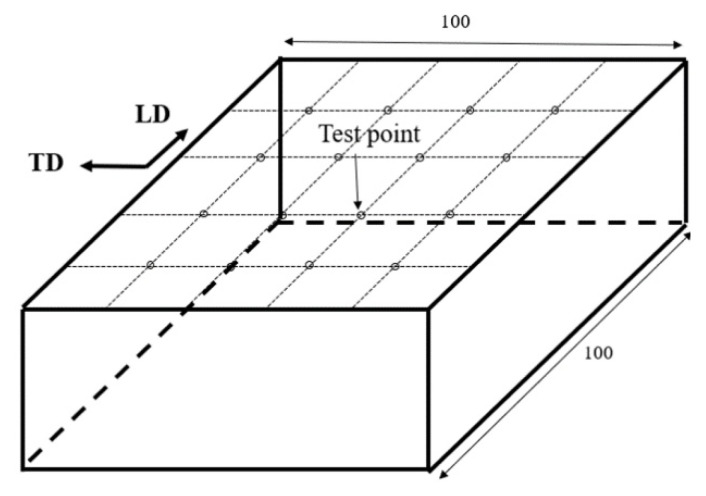
Diagram of the 2A14 aluminum alloy specimens and test points.

The four sizes of specimens were of thick aluminum alloy plates, and the surface was marked with 5 × 5 grids with equal spacing. The intersection of grid lines was selected as the location of measurement points, and there was a total of 16 measurement points on the surface of each specimen. In this experiment, the parallel rolling direction (LD) was defined as the X direction and transverse direction (TD) was defined as the Y direction; the residual stress in the X direction was measured at each point, as shown in Figure 1.

To avoid the influence of mechanical processing on the experiment, the specimens were divided into three groups and subjected to different solutions and aging processes. The solution process of the three groups of specimens was at 500 °C for 4 h, and the quenching medium was water at 60 °C. The three groups of specimens were aged at 170 °C for 2 h, 4 h, and 6 h and then cooled to room temperature.

### 2.2. Characterization of Residual Stress

#### 2.2.1. X-ray Diffraction

**Figure 2:**
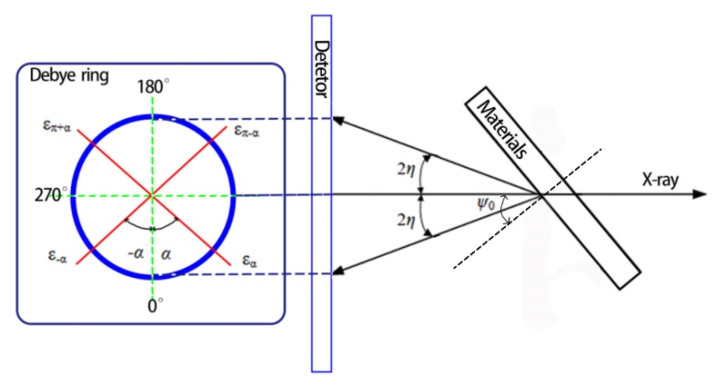
The distortion of the Debye ring, collected by an area detector.

In this experiment, the cosα method of XRD and synchrotron radiation technology [19] were used to measure the residual stress on the specimen surface. The cosα method is based on Debye ring diffraction information for stress analysis and calculates the residual stress through Debye ring distortion (which is still lattice distortion, in essence); the distortion of the Debye ring, collected by an area detector, is shown in Figure 2. According to the Bragg equation and elastic mechanics equation, which can be defined as:

(1) $$a_{1}=\frac{1}{2}\left\{\left(\varepsilon_{\alpha }-\varepsilon_{\pi +\alpha }\right)+\left(\varepsilon_{-\alpha }-\varepsilon_{\pi -\alpha }\right)\right\},$$

the strain *ε_α_* at the Debye ring can be effectively used to estimate the residual stress in the specimen [20,21]:

(2) $$\sigma =-\frac{E}{1+v}\frac{1}{\sin^{2}\psi_{0}}\frac{1}{\sin^{2}\eta }\left(\frac{\partial a_{1}}{\partial \cos\alpha }\right).$$

The meaning of each variable is:

$\psi_{0}$: the angle between the incident X-ray beam direction and the normal direction to the specimen surface;

v: Poisson’s ratio of materials;

α: the angle at the Debye ring;

E: the elasticity modulus of materials;

η: the diffraction angle between the reflection line and input X-ray.

The XRD cosα method uses X-ray diffraction equipment (XRD, μ-X360, Pulstec, Binsong, Japan) for stress measurement. The anode target is the Cr target. The X-ray tube power is 30 KV/1.5 mA, the collimator is 1 mm, the diffraction peak is Al (311), the test angle is 25°, and the measurement time is 5 min.

#### 2.2.2. Synchrotron Radiation X-ray Diffraction

The stress measurement of synchrotron radiation used in this experiment is based on sin^2^ψ, which was first put forward by E. Macherauch [22]. It is based on the measurements of the lattice parameters, determined from a fixed hkl Bragg reflection but at a different tilt angle, ψ [23]. According to the Bragg equation and elasticity equation, the residual stress calculation formula is derived as follows, which is based on 2θ methods [24].

(3) $$\sigma =-\frac{E}{2\left(1+v\right)}\;\cot\theta_{0}\;\frac{\pi }{180}\;\frac{\partial \left(2\theta_{\psi }\right)}{\partial \left(\sin^{2}\psi \right)}$$

The meaning of each variable is:

$\theta_{\psi }$: the angle between the X-ray incident beam and the normal sample surface;
ψ: the angle between the measured strain direction and the normal specimen surface;
v: Poisson’s ratio of materials;
E: the elasticity modulus of materials;
$\theta_{0}$: the diffraction angle without stress.

The calculation was made by taking 2θ as the ordinate and sin^2^ψ as the abscissa, the slope obtained from this figure is the residual stress on the surface of the specimen.

Synchrotron radiation sin^2^ψ testing was conducted at the 1W1A diffraction station of the Beijing Synchrotron Radiation Facility. The main experimental equipment of the diffraction station is a five-circle diffractometer, with a beam energy of 8 Kev and a spot size (H × V) of 0.7 mm × 0.4 mm. In accordance with the measurement standard, the diffraction energy selected for this experiment was 8 Kev, with λ = 0.1544 nm, and the diffraction angle on this occasion was about 78.4°. The experimental parameters were selected according to EU standard EN5305-2008 for the determination of residual stress, and CHN standard GB/T7704-2008 for X-ray diffraction. When the measurements were taken, the rotation step of the diffractometer was set at 0.02° and the integration time of the detector was set at 2 s.

### 2.3. Characterization of Microstructure and Morphology

Electron backscattered diffraction (EBSD), as used in field emission scanning electron microscopy (FSEM, EVO25, Zeiss, Oberkochen, Germany), was employed to scan the tested areas of the specimens after different heat treatments, and the grain size and orientation of the specimens were measured. The voltage and working distance were 15 kV and 10 mm, respectively. First, the aluminum alloy was processed into 10 mm × 10 mm × 3 mm specimens and the test surface of the specimen was polished, cleaned using ultrasound, and polished via electrolytic polishing. The electrolyte was prepared by mixing 10% perchloric acid and 90% ethanol. After electrolytic polishing, the specimen was immediately cleaned with alcohol, then the specimen was blown dry for use. The EBSD data and Back-Scaterred Electron images were analyzed using OIM Analysis software. The microstructure of the aluminum alloy was measured with an optical microscope (OM) (Axio Lab A1, Zeiss, Oberkochen, Germany).

## 3. Results and Discussion

### 3.1. Structural Characterization of Aluminum Alloy after Different Solutions and Aging Treatments

#### 3.1.1. Structural Characterization

**Figure 3:**
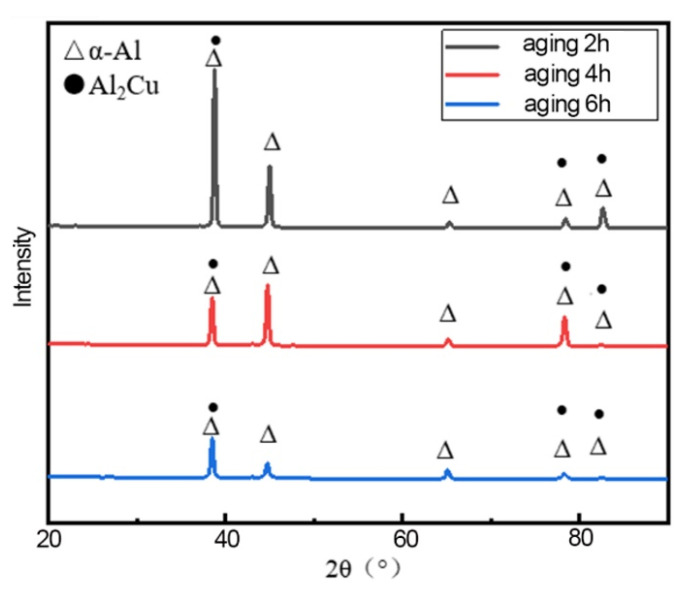
The X-ray diffraction patterns of the 2A14 aluminum alloy (50 mm) surface at different aging stages.

XRD was used to characterize the three groups of specimens, and the effect of heat treatment conditions on the phase composition of 2A14 aluminum alloy was analyzed. The results are shown in Figure 3. The main precipitated phases in 2A14 aluminum alloy are the S phase (Al_2_CuMg) and θ phase (Al_2_Cu). It can be seen that the main phases are the Al_2_Cu phase and α-Al matrix in the XRD patterns in the specimens with three different heat treatments; no diffraction peak for Al_2_CuMg can be observed in Figure 3, which indicates that the content of the S phase is not high enough to be detected by XRD [25].

#### 3.1.2. Metallographic Structure

**Figure 4:**
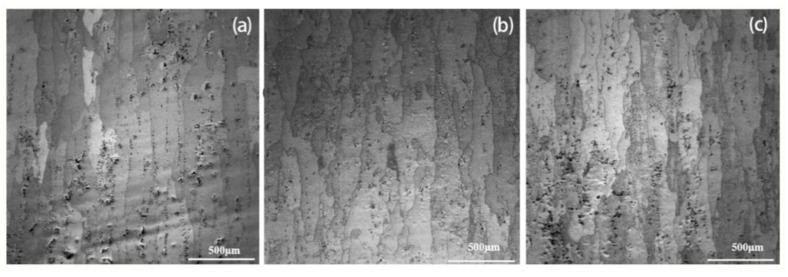
The surface microstructure of the 2A14 aluminum alloy (50 mm): (**a**) aging for 2 h; (**b**) aging for 4 h; (**c**) aging for 6 h.

The microstructure of the 50-mm-thick 2A14 aluminum alloy after different solution aging treatments, which was investigated using an OM, is shown in Figure 4. It can be seen that under different heat-treatment conditions, most of the grains of the alloy are fibrous, showing a large aspect ratio along the rolling direction, and the grain boundaries are rough and serrated. The grain size of the alloy is not evenly distributed along the depth direction, exhibiting fine surface grains and coarse internal grains. With the increase in aging time, the grain size of the specimen does not change obviously, and the black second phase begins to precipitate in the Al matrix. With the increase in aging time, the density of the second phase gradually increases. The reason for this is that the surface body has large amounts of free energy, a high recrystallization driving force, and a large number of crystallized nucleation, while the inner body has a small amount of free energy and a low recrystallization driving force, which is mainly manifested as grain growth, diffusion, and the dislocation movement of materials at the grain boundaries [26].

#### 3.1.3. Grain Orientation and Texture

**Figure 5:**
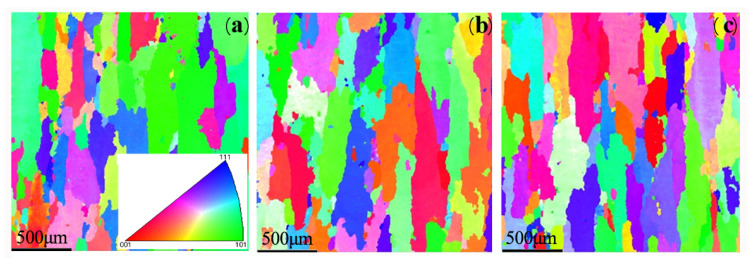
EBSD grain morphology of 2A14 aluminum alloy (50 mm) under different processes: (**a**) aging for 2 h; (**b**) aging for 4 h; (**c**) aging for 6 h.

**Figure 6:**
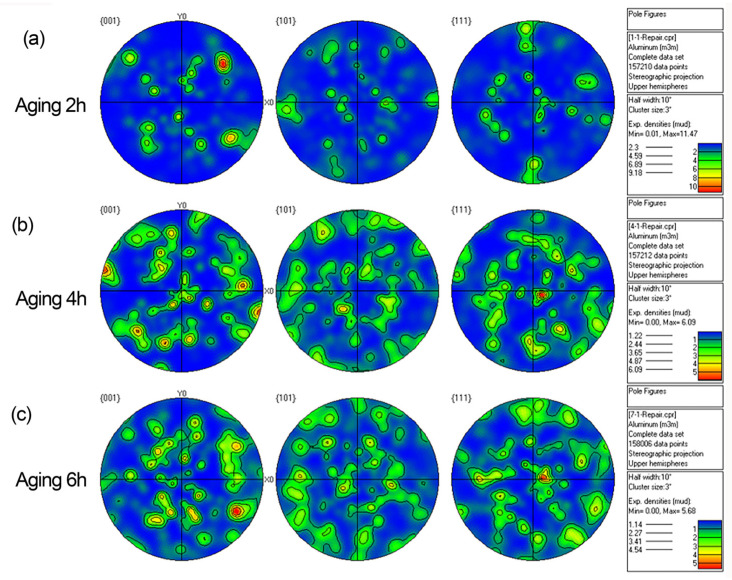
Reverse polarity diagram of a 2A14 aluminum alloy (50 mm) specimen after different processes: (**a**) aging for 2 h; (**b**) aging for 4 h; (**c**) aging for 6 h.

An EBSD test was conducted for aluminum alloy materials under different solution processes, to analyze the effect of aging on grain distribution and texture. The distribution of the grain texture is shown in Figure 5 and Figure 6. After the solution treatment for the 2A14 aluminum alloy, the grain shape of the 2A14 aluminum alloy was mainly elongated along the rolling direction, with an uneven size. It can be seen from these figures that the massive solution from the second phase was dissolved into the lattice and formed a supersaturated solution after the solution treatment was applied to the sample, causing severe lattice distortion; as a result, the grain size was the largest at this time. With the increase in aging time, part of the second phase began to precipitate, with weaker lattice distortion. After aging for 4 h at 170 °C, the grain size of the thick plate surface increased with an uneven grain size distribution, and some textures were eliminated. In addition, there were occasionally large anisometric grains. After aging for 6 h, the grain size on the surface was large but showed an uneven size distribution; there were many textures with different orientations in three directions. It can be seen from the pole diagram that the texture density increased with the increase in aging time and the samples at all depths did not have a texture indicating strong orientations.

### 3.2. Characterization of the Residual Stress in Aluminum Alloy after Different Solution Aging Treatments

#### 3.2.1. Characterization of the Residual Stress by X-ray Diffraction

**Figure 7:**
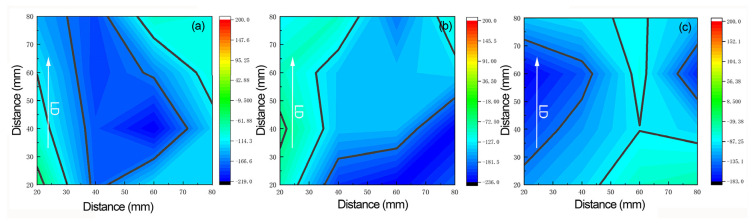
Surface residual stress distribution of 2A14 aluminum alloy (50 mm) under different aging conditions: (**a**) aging for 2 h; (**b**) aging for 4 h; (**c**) aging for 6 h.

The measured residual stresses on the surface of the 2A14 aluminum alloy plate and the distribution nephogram of the residual stresses in the parallel rolling direction of three groups of samples are shown in Figure 7. Figure 7a–c represents the stress distribution of solution treatment, with aging for 2 h, aging for 4 h, and aging for 6 h, respectively. After solution treatment and aging the samples for 2 h, the residual stresses on the surface of the thick plate showed a distribution trend of external tension and internal compression, with a maximum residual stress of −190 MPa and the compressive stresses concentrated in the center. After solution treatment and aging for 4 h, the residual stresses at the center were partially released, and the maximum residual stress became −152 MPa, with a decrease of 20%. In addition, the concentration of the compressive residual stresses was also relieved, with the stresses in the edge area transformed into compressive stresses. It can be seen that the change in residual stresses was positively related to the aging time. Compared to the results of aging for 2 h and 4 h, the release effect of the residual stresses was even better after aging for 6 h. The maximum stress value at the center was only −120 MPa, with a decrease of 36.8%. When the temperature was 170 °C, the degree of lattice distortion in the alloy was restored, to a certain extent. The residual stress state of the entirety of the thick plate was then redistributed, with part of the stresses being released, achieving a balance between the stresses in each part.

**Figure 8:**
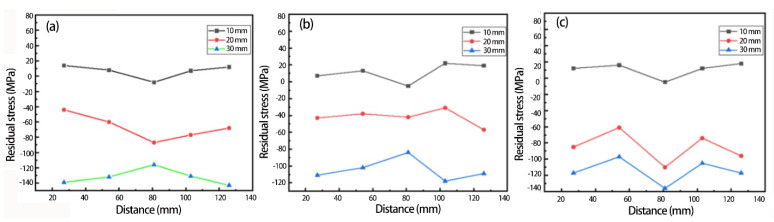
Residual stress under different aging conditions (**a**) aging for 2 h; (**b**) aging for 4 h; (**c**) aging for 6 h.

The measurement results of residual stresses on the surface of aluminum alloy samples of different thicknesses in each group of processes along the rolling direction are shown in Figure 8. It can be seen that the residual stress on the surface of the plates undergoing each process was significantly increased with the increase in the thickness of the aluminum alloy plates. Among these samples, the residual stress level in the 10-mm-thick samples was the lowest, at less than 20 MPa, and was evenly distributed. After aging for 2 h, the residual stress level of the 30-mm-thick samples reached about −140 MPa, with the residual stress at the center being slightly lower than that at both ends. When the soaking time of the aging process was increased, the residual stress level on the surface of the 30-mm-thick samples decreased slightly to −120 MPa, but the residual stress level on the surface of the 10-mm-thick samples did not change significantly. Due to the different temperature gradients on the surface and inside of the aluminum alloy, large residual stresses were generated during the solution process. Since there was a small difference in heat dissipation speed between the surface and the interior of the thinner plates and in the deformation between the surface and the interior during rapid cooling, the residual stress level decreased with the decrease in thickness. Due to the difference in internal and external deformation of aluminum alloy samples with a thickness of 30 mm, a high level of residual stress was introduced onto the surface of the samples. During the aging process, the aluminum alloy structure gradually stabilized, and the lattice distortion decreased; therefore, the high level of residual stresses introduced during the solution treatment also began to reduce. When the soaking time of the aging process increased from 2 h to 6 h, the residual stress level in the 30-mm-thick aluminum alloy plate decreased by about 14.3%, but the effect of the aging process on reducing the residual stress level was relatively insignificant for the 10-mm-thick samples because the residual stress level introduced during the solution treatment was relatively low.

#### 3.2.2. Characterization of Residual Stress by Diffraction Using Synchrotron Radiation

The measurement of residual stresses of the 50-mm-thick plates was conducted using the sin^2^ψ method, via synchrotron radiation technology. The diffraction data of the samples measured in the synchrotron radiation experiment after the different aging treatments were processed, and all the diffraction peaks that were obtained were fitted. The peak fitting was conducted using the Gauss profile [23,27].

**Figure 9:**
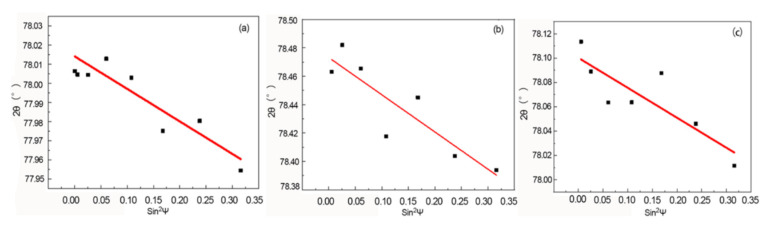
The linear fit of the specimen test points: (**a**) aging for 2 h; (**b**) aging for 4 h; (**c**) aging for 6 h.

The sin^2^ψ value was calculated by using the 2θ values of each test point of the three groups of samples that were obtained by fitting. Since the fitting method for the data of each measured point was the same, the fitting results of only one of the measured points were represented for each process. The curve for the 2θ-sin^2^ψ relationships of the samples is shown in Figure 9. The red line and black points represents fitting line and experiment pionts, respectively. Vibration occurred at the fitting point. Combined with EBSD and metallographic photographs, it can be seen that the texture in the sample will make the fitting point vibrate, which will affect the accuracy of fitting to a certain extent, but the linear trend of the first fitting result was good, so that the vibration has little impact on the measurement results.

**Figure 10:**
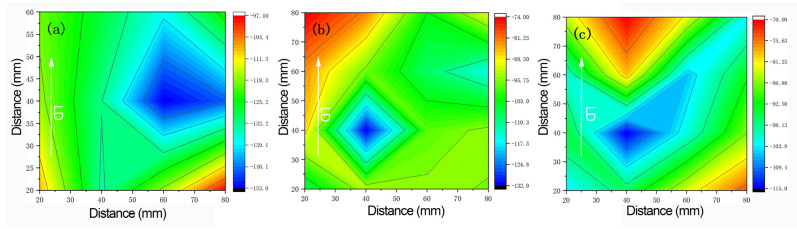
Surface residual stress distribution under different aging conditions: (**a**) aging for 2 h; (**b**) aging for 4 h; (**c**) aging for 6 h.

After statistical fitting on all measurement results under each process condition, the calculation of the residual stress value of each point was conducted to make a nephogram, which is shown in Figure 10. After aging for 2 h (Figure 9a) for the residual stresses on the surface of the 2A14 thick aluminum alloy plates, the residual stresses near the surface were identified as compressive stresses, with a maximum value of −153 MPa at the center of the thick plates, and the edge stress level was relatively low, at about −100 MPa. After the solution treatment and aging for 4 h (Figure 9a), the residual stresses of the sample were released to a certain extent, but the trend of high compressive stresses at the center was still maintained, with a maximum value of about −125 MPa. After aging for 6 h (Figure 9a), the stress in the central area of the sample decreased to −115 MPa, with a decrease of 24.8%.

Compared with the distribution of residual stresses on the surface, which was measured using XRD in the laboratory, the stress level, which was measured using XRD from synchrotron radiation sources, decreased slightly, while the stress state remained unchanged in the form of compressive stress, but the stress concentration area at the center was greatly decreased. This occurred because the rate of heat transfer was fast and was not limited by the volume on the surface; however, some stresses could be released through microdeformation. As a result, the effect of this reduction on the residual stresses was more obvious during the aging process.

By comparing the trends of the residual stresses and microstructures under different technological conditions, it can be concluded that the residual stresses on the surface of the 2A14 thick aluminum alloy plates were compressive stresses, with the largest compressive stress level found in the center. The surface residual stresses of the samples were great after the solution treatment, with the lattice distortion and increasing grain size caused by the massive S-phase solution; as a result, the residual stress level increased. After aging at 170 °C for 4 h and 6 h, a large amount of the S phase began to precipitate. The grain orientation was now no longer single; with the grain size slightly reduced, the lattice distortion was reduced, and the residual stresses were released. Therefore, the stress level decreased accordingly, in line with aging time.

Comparing the XRD measurement results using laboratory testing and synchrotron radiation, we established that the stress distribution obtained by the two residual stress calculation methods, cosα and sin^2^ψ, under different process conditions was consistent; namely, that the stress distribution on the surface of the samples was of the compressive stress type, and the residual stresses decreased significantly with an increase in aging time. However, the stress value measured via the traditional XRD method was higher than that measured with the synchrotron radiation method, which was due to the larger spot diameter and lower resolution of a traditional X-ray and the larger fluctuation of measurement data. Among other advantages, the traditional XRD method has high measurement efficiency, and there is no need to change the incidence angle, with 500 diffraction data values obtained for the numerical fitting of residual stress by each X-ray incidence. Conversely, the synchrotron radiation diffraction technology has a smaller beam, higher resolution, and a higher signal-to-noise ratio.

## 4. Conclusions

After solution aging of 2A14 thick aluminum alloy plates, the grains became fibrous and were distributed unevenly. As the aging time increased, the θ phase gradually precipitated from the matrix, although the grain size did not change significantly. During aging, there is no strong texture; there is only a randomly distributed weak texture, indicating that there is no obvious preferred orientation. After solution aging, the surface residual stress is compressive stress, and the peak stress is in the central area of the plate. The peak value measured via the X-ray method is −236 MPa, while that measured by the synchrotron radiation method is −153 MPa. After solution treatment, due to the uneven heat dissipation on the surface and inside of the plate, the internal cooling shrinkage of the specimen was uneven, resulting in serious residual stress. After aging, the strengthening phase dispersed and precipitated; the lattice distortion decreased, and the residual stress decreased with the increase in aging time. A wider range of ψ and a higher number of ψ stations for use in the sin^2^ψ method can be selected to measure the residual stress, so as to improve measurement accuracy. However, the cosα method uses a single exposure, and still needs to be optimized, the range of ψ is not broad enough and will cause a large measurement error.

## Figures and Tables

**Table 1:** Chemical composition of 2A14 aluminum alloy (wt %).

Cu	Mg	Mn	Si	Fe	Zn	Al
4.32	0.64	0.84	0.85	0.29	0.08	Bal.

## Data Availability

The raw/processed data required to reproduce these findings cannot be shared at this time as the data also forms part of an ongoing study.
